# TiO_2_-Doped Electrospun Nanofibrous Membrane for Photocatalytic Water Treatment

**DOI:** 10.3390/polym11050747

**Published:** 2019-04-26

**Authors:** Miren Blanco, Cristina Monteserín, Adrián Angulo, Ana Pérez-Márquez, Jon Maudes, Nieves Murillo, Estíbaliz Aranzabe, Leire Ruiz-Rubio, Jose Luis Vilas

**Affiliations:** 1 Unidad de Química de superficies y Nanotecnología, Fundación Tekniker, Iñaki Goenaga 5, 20600 Eibar, Spain; cristina.monteserin@tekniker.es (C.M.); adrian.angulo@tekniker.es (A.A.); estibaliz.aranzabe@tekniker.es (E.A.); 2 Division Industria y Transporte, TECNALIA, P Mikeletegi 7, E-20009 Donostia-San Sebastian, Spain; ana.perez@tecnalia.com (A.P.-M.); jon.maudes@tecnalia.com (J.M.); nieves.murillo@tecnalia.com (N.M.); 3 Grupo de Química Macromolecular (LABQUIMAC) Dpto. Química-Física, Facultad de Ciencia y Tecnología, Universidad del País Vasco (UPV/EHU), 48940 Leioa, Bizkaia, Spain; joseluis.vilas@ehu.eus; 4 BCMaterials, Basque Center for Materials, Applications and Nanostructures, UPV/EHU Science Park, 48940 Leioa, Spain

**Keywords:** membrane filtration, polymeric nonwoven nanofibres, needle-free electrospinning, photocatalytic activity, TiO_2_ nanoparticles, decontamination, chemical hazards, bacteria

## Abstract

This work has been focused on the one-step fabrication by electrospinning of polyamide 6 (PA6) nanofibre membranes modified with titanium dioxide nanoparticles (TiO_2_), where these TiO_2_ nanoparticles aggregates could induce a photocatalytic activity. The main potential application of these membranes could be the purification of contaminated water. Thus, it is important to analyse the contaminant degradation capability since in these membranes this is based on their photocatalytic activity. In this work, the effect of the photocatalysis has been studied both on the degradation of an organic model contaminant and on the removal of *Escherichia coli* and other coliform bacteria. As a result, it was observed that these membranes present excellent photocatalytic activity when they are irradiated under UV light, allowing a 70% reduction of an organic model pollutant after 240 min. In addition, these membranes successfully removed *Escherichia coli* and other coliform bacteria in artificially inoculated water after 24 h of contact with them. Moreover, the stand-alone structure of the membranes allowed for the reusing of the immobilized catalyst. The experimental evidence indicated that developed nanofibre membranes are a fast and efficient solution for polluted water decontamination based on photocatalysis. Their use could contribute to guarantee a fresh water level and quality, mitigating the water scarcity problem worldwide.

## 1. Introduction

Water covers 70% of our planet, however freshwater represents the 6% of the world’s water, and two-thirds of that is in frozen glaciers, therefore fresh water is a scarce good. Moreover, due to the rapid development of manufacturing technologies and inefficient environmental policy, wastewater is becoming more contaminated and difficult to process [1]. Considering the difficulties to access to clean water supplies that inhabitants of many countries face all-over the world, the water protection and the contamination prevention or remediation is a global problem that needs to be solved urgently. For these reasons, there is a considerable interest in the development of techniques to restore the water quality. There are several physicochemical methods such as filtration, chemical and electrochemical oxidation or ozone treatment, among others, used to resolve this problem. Between them, heterogeneous photocatalysis is considered an inexpensive, viable and effective alternative or complimentary method for water and wastewater treatment [2]. In a photocatalytic process, the photons that possess higher energy than the band-gap energy excite valence band electrons, enhancing the subsequent reaction of photocatalysts with different molecules [3]. The illumination with sufficient energy for the catalyst active sites produces positive holes (h^+^) in the valence band and generates electrons (e^−^) in the conduction band. The positive holes oxidise either H_2_O or organic pollutants to induce hydroxyl radicals [4]. Summarising, a catalyst harnesses the ultraviolet radiation (UV) (<385 nm) [5] and non-selective and highly reactive species like hydroxyl radicals (·OH) are generated via specific chemical reactions in aqueous mediums [6]. Hydroxyl radicals can destroy a wide variety of pollutants, such as organic acids, pesticides, hormones, dyes, microorganisms and even inorganic compounds [7].

TiO_2_ nanoparticles are one of the most promising and widespread photocatalysts as: (1) the nanometric size gives them a large specific area, increasing their reactivity; (2) they are commercially available at large scale, abundant and thus inexpensive; (3) TiO_2_ is chemically stable and possesses a high capability to catalyse degradation processes via the disruption of molecular bonds. However, there is some concern about the negative impact of nanoparticles on human health and the environment that prevent their use without being part of a composite material. In addition, the recovery of the photocatalysts from the medium after the water remediation is still a challenge, and it could limit the applicability of the photocatalytic technique. In this framework, the incorporation of the photocatalysts, TiO_2_ nanoparticles in this case, anchored on a substrate could be considered a highly promising alternative to overcome the recovery or other similar issues [6,8,9,10,11,12,13].

The use of TiO_2_ coated on a surface presents low catalytic activity due to the poor dispersion of the catalytic particles and limited interactions between the contaminant and the catalyst [14,15]. Several studies described that catalysts could be more effective when they are attached to an adsorbent surface such as powdered activated carbon [6], clays [16], expanded perlite [17] or SiC foams [18]. In this context, an electrospinning technique is a simple and cost-effective method to manufacture high surface area materials with high porosity and small pore sizes from a large collection of materials, from polymeric to ceramics, including complex composites [2,19,20,21]. In fact, it is possible to find an air-filtering solution based on polymeric electrospun nanofibres, but their use in water applications is still under development. Consequently, in the last decade, significant efforts regarding the formulation and optimisation of materials incorporating TiO_2_, ZnO, carbon nanotubes or graphene, among others [15,22,23,24,25,26,27], have been made. There are different ways of including these nanoadditives in the membranes, mainly the direct addition of nanomaterials in the solution used as precursor of the electrospun fibres, and the addition of the materials on membrane surfaces by their immersion in a reactive formulation with the nanoadditives. Between them, the direct incorporation of nanoadditives to the nanofibre material seems to improve the efficiency of the final filters as it is avoids the blocking of the pores due to a subsequent coating of the filters [15]. 

For the present work, authors have studied the use of polyamide 6 (PA6), a polymer widely used in water treatment, as a support for the TiO_2_ nanoparticles in order to develop micro/nanoporous hybrid nonwoven membranes with excellent photocatalytic activity under UV light irradiation. These membranes could be used as a one-step stand-alone membrane on filtration devices for water purification from organic contaminants, but also for the efficient removal of bacteria. Currently, the obtaining of antimicrobial membranes is being addressed through the inclusion of silver nanoparticles or silver-based compounds in the membranes, and the use of TiO_2_ for photocatalysis and antimicrobial properties has been poorly explored. The morphology and areal weight density of the electrospun nanofibres membranes have been characterised using scanning electron microscopy (SEM) and weight measurements, respectively. The presence of nanoparticles in the nanofibres have been checked using SEM, Fourier transform infrared (FTIR) and thermogravimetric analysis (TGA). In addition, the photocatalytic activity under UV radiation against a model organic pollutant, Remazol Black B (C.I. Reactive Black 5), and against *Escherichia coli* (*E. coli*) and other coliform bacteria (ISO 7704: 1985), has been also evaluated. 

## 2. Materials and Methods 

### 2.1. Materials

For preparation of the nanofibre membranes, polyamide 6 has been selected as the polymeric material as it is widely used in water treatment applications and presents a high mechanical performance, with it being able to easily handle membranes and its direct use as filter in the decontamination process. A PA6 Ultramid B24 N03 purchased by BASF (Tarragona, Spain) has been employed. Acetic acid and formic acid employed for the PA6 solution have been purchased from Panreac AppliChem, Barcelona, Spain. Titanium dioxide of analytical grade of purity (Aeroxide® P25 TiO_2_, < 25 nm) has been obtained from Evonik Corporation (Essen, Germany). Sodium dodecyl sulfate (SDS, ≥98.5%, Sigma-Aldrich, St. Louis, MO, USA) and poly(ethylene glycol) (PEG, number-average molecular weight (*M*_n_) = 400 g·mol^−1^, Sigma-Aldrich) have been employed as surfactants for TiO_2_ nanoparticles dispersion. As a model organic pollutant, a commercial diazo reactive dye containing two vinyl sulfones as reactive groups, Remazol Black B (C.I. Reactive Black 5) were employed. This dye has an empirical formula C_26_H_21_N_5_Na_4_O_19_S_6_ and molecular weight 991.8 g/mol, and has been purchased from Acros Organics (Morris, NJ, USA). All chemicals are analytical grade substances and have been used without further purification. 

### 2.2. PA6 and TiO_2_ Modified PA6 Electrospun Nanofibres Membranes Preparation

For the development of PA6 electrospun nanofibres membranes, a 12 wt% of polyamide 6 was dissolved in a mixture 2:1 of acetic acid:formic acid via mechanical stirring vigorously (1200 rpm) for 2 h at 80 °C. The nanofibres have been produced using a multijet electrospinning setup Nanospider™ from Elmarco (Liberec, Czech Republic) [15,28]. This device allowed for producing large uniform nanofibrous structures that were easy to scale to higher dimensions. Manufacturing conditions of the membranes were optimized to achieve homogeneous diameter distribution and to avoid defects presence (beads, excess solvent, etc.). These defects could affect to the physical adsorption capability of the membranes. As a result, highly homogeneous nonwoven membranes were obtained without defects. All nonwoven nanofibrous membranes were obtained at 23 ± 2 °C and 50 ± 5% relative humidity (RH). In addition, the spinning rate was established at 6.7 rpm and the distance between the collector and the cylinder was set at 170 mm. The voltage was adapted until it achieved a stable process, in this case 75 kV. The stand-alone structures were electrospun onto an antistatic polypropylene substrate. Following previous optimization [28], in order to avoid viscosity variations due to the solvent evaporation, the electrospinning duration time was 60 min.

For the manufacturing of the veils modified with TiO_2_, the nanoparticles were added to the PA6 solution described above, obtaining a system with 25% by weight of nanoparticles with respect to PA6. The stabilisation of the nanoparticles required the addition of SDS as a surfactant in a 1:1 weight ratio with respect to the nanoparticle weight and the use of 30 min of ultrasound (US Sonics VCX series, Sonic and Materials Inc, Newton, MA, USA) with a power of 750 W and an amplitude of 30%). The procedure for the selection of the surfactant can be found in the Supporting Information (Figure S1, Table S1). Electrospinning process parameters remained similar to the employed parameters for obtaining unmodified PA6 nanofibres.

### 2.3. PA6 and TiO_2_ Modified PA6 Electrospun Nanofibres Membranes Characterization

Scanning electron microscopy (SEM) was employed to analyse the morphology and topography of the nanofibrous materials. A Carl Zeiss SMT Ultra Gemini-II microscope (Carl Zeiss, Thornwood, NY, USA) was employed. Samples were analysed without being coated. The areal weight density of the electrospun nanofibres membranes was determined by weighing a 10 × 10 cm area in a balance with an accuracy of 0.0001 g. To determine the presence of TiO_2_ nanoparticles on electrospun nanofibres, a Fourier transform infrared spectroscope (FTIR 4700 JASCO, Easton, PA, USA) was employed. Spectra were obtained using an attenuated total reflection accessory, PIKE GladiATR (Pike Technologies, Madison, WI, USA). Twenty scans were averaged for each sample in the range of 400 cm^−1^ to 4000 cm^−1^ with a resolution of 4 cm^−1^. The presence of TiO_2_ nanoparticles on electrospun nanofibres was also confirmed using thermogravimetric analysis (TGA) by using a SDT Q600 analyzer from TA instruments, New Castle, DE, USA. The analysis was performed from room temperature to 800 °C under a nitrogen atmosphere (100 mL/min) with a heating rate of 10 °C/min. 

### 2.4. Photocatalytic Performance of Electrospun Nanofibres Membranes

To determine the photocatalytic performance of the TiO_2_ modified membranes and its possible use for water treatment processes, the effect of the membranes in the decomposition of an organic model pollutant was studied. In addition, the capability of these membranes to remove *E. coli* and other coliform bacteria was analysed. For comparison purposes, the performance of PA6 electrospun nanofiber membranes was also tested and used as a reference. 

A synthetic diazo reactive dye containing two vinyl sulfones as reactive groups, Remazol Black B (C.I. Reactive Black 5) was used as a model contaminant to study the absorption and photocatalytic activity of the electrospun nanofibres membranes. Remazol Black B was selected because it is widely used in the textile industry due to its high chemical stability and it does not entirely fix on the fabrics, polluting water sources [29]. Moreover, once azo dyes enter wastewater, they become more stable and are more difficult to biodegrade because of their complex chemical structures [30]. A stock solution with 3 mg/L in distilled water was prepared. Samples of 4 × 4 cm were cut and two pieces were immersed into 200 mL of the solution owing to Teflon frameworks. The membrane samples were kept for 60 min in the dark to reach an adsorption–desorption equilibrium, and then they were irradiated by two UV lamps with an emission maximum at 365 nm (UV lamp Vl-6-L, from Vilber Lourmat (Collégien, France) for 4 h. The photocatalytic process was monitored by following the intensity decrease of the dye absorbance at 597 nm with respect to the irradiation time using spectroscopy. UV–visible spectra at the wavelength range 380–1000 nm were recorded with a Perkin Elmer (Waltham, MA, USA) Lambda 950 spectrophotometer. An aliquot was taken every 15 min. Initially, the stability of Remazol Black B under the UV radiation was observed.

For testing the performance of electrospun nanofibres membranes in the photocatatytic removal of *E. coli* and other coliform bacteria, first, deionized water was artificially inoculated with 1 μL of sewage water to introduce a concentration of *E. coli* and other coliform bacteria suitable for measurement. *E. coli* and the other coliforms were used in these experiments due to their widespread use as a faecal indicator and its resistance to the bactericidal effects of light irradiation relative to other bacteria [31]. Chromogenic coliform agar (CCA) was employed for the detection of *Escherichia coli* and coliform bacteria in this medium. Coliforms were distinguished via the production of β-galactosidase, an enzyme that reacts with the chromogenic substrate to generate a pink to red precipitate. *E. coli* possesses β-galactosidase and 94–97% were also positive for β-glucuronidase. The presence of the enzyme was revealed by a blue coloration. The simultaneous action of the two enzymes gave the colonies of *E. coli* a purple colour. CCA Microinstant ® kits (Scharlab, Barcelona, Spain) that were employed in this study complied with the formula described in the ISO 9308-1:2014 standard. An area of 4 × 4 cm of PA6 and TiO_2_-modified PA6 electrospun nanofibres samples were cut and placed in contact with 100 mL of the artificially inoculated water for 24 h, with and without UV light. After this time, the water was filtered through a membrane of 0.45 μm pore diameter, validated according to ISO 7704: 1985. The membrane used in this filtration process was inserted face up on a dish containing the CCA. The dishes with membranes were incubated at 36 ± 2 °C. A photograph of the membranes was taken after 24 h and 4 days of incubation.

## 3. Results and Discussion

### 3.1. PA6 and TiO_2_-Modified PA6 Electrospun Nanofibres Membranes Characterization

**Figure 1:**
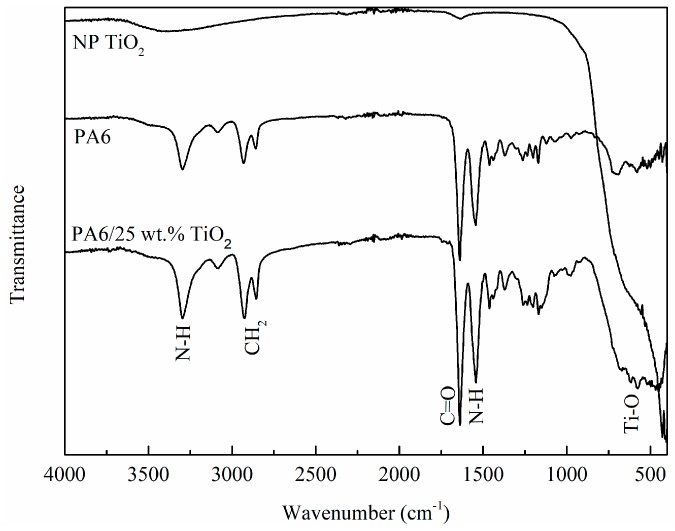
FTIR spectra of TiO_2_ nanoparticles, PA6 electrospun nanofibres and PA6/25 wt.% TiO_2_ electrospun nanofibres.

Physicochemical characterization of the membranes was carried out by using FTIR and TGA analysis. Initially, FTIR-ATR was used to characterise the membranes (Figure 1). For both electrospun nanofibre membranes, the characteristic polyamide absorption bands were observed at 3300 cm^−1^ and were assigned to the N–H stretch of the amide group. The vibrations for C=O stretching appeared at 1680 cm^−1^ and asymmetric and symmetric CH_2_ stretching vibrations were observed at 2919 and 2850 cm^−1^, respectively [32]. A band appeared around 1550 cm^−1^, which was ascribed to the deformation of the N–H bond. The vibrational modes of amide were observed at 1270 cm^−1^ (C–N stretching) and 620 cm^−1^ (C–N deformation), respectively. In the spectra corresponding to the TiO_2_ modified electrospun nanofibres, an increase in the contribution of the band centered at 550 cm^−1^ was observed, which corresponds to Ti–O bonds [33], indicating the successful incorporation of the TiO_2_ on the PA6. However, similarly to other reported studies, the incorporation of TiO2 nanoparticles on the nanofibers showed no significant changes in the FTIR spectrum due to the physical interactions between the matrix and the nanoparticles not being strong enough to considerably distinguish the characteristic peaks [34]. The spectra of the TiO_2_ nanoparticles was also included for clarification. 

**Figure 2:**
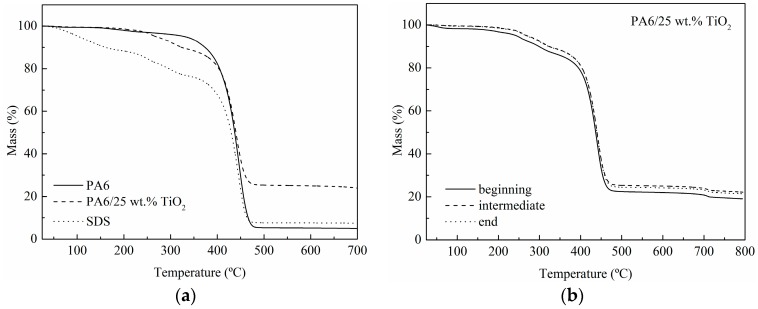
TGA thermograms of: (**a**) membranes made of PA6 electrospun nanofibres and PA6/25 wt.% TiO_2_ electrospun nanofibers, and (**b**) three different areas of the PA6/25 wt.% TiO_2_ electrospun nanofibres.

The thermogravimetric analysis of PA6 electrospun nanofibres and PA6/25 wt.% TiO_2_ electrospun nanofibres membranes under nitrogen atmosphere (Figure 2a) showed that both membranes had a significant mass loss at temperatures close to 400 °C corresponding to the degradation of the polymeric matrix. However, the nanofibers modified with 25% TiO_2_ presented an unexpected fall between 250 and 400 °C. This fall was ascribed to the presence of SDS used as a surfactant in the stabilisation of the nanoparticles in the precursor solution during the membrane preparation. The TGA curve of the SDS was also included in Figure 2a for clarification. The residue after degradation of the organic part for PA6/25 wt.% TiO_2_ electrospun nanofibres was approximately 22–25% by weight, close to the amount of nanoparticles initially included during membrane preparation. Moreover, TGA was employed to test the homogeneity of the PA6/25 wt.% TiO_2_ electrospun nanofibres. The analysis of three different areas of the membrane (the beginning, the end and an intermediate zone) showed that the concentration of nanoparticles was similar along the membrane, so the preparation process employed was shown to be adequate to achieve homogeneous membranes (Figure 2b).

**Figure 3:**
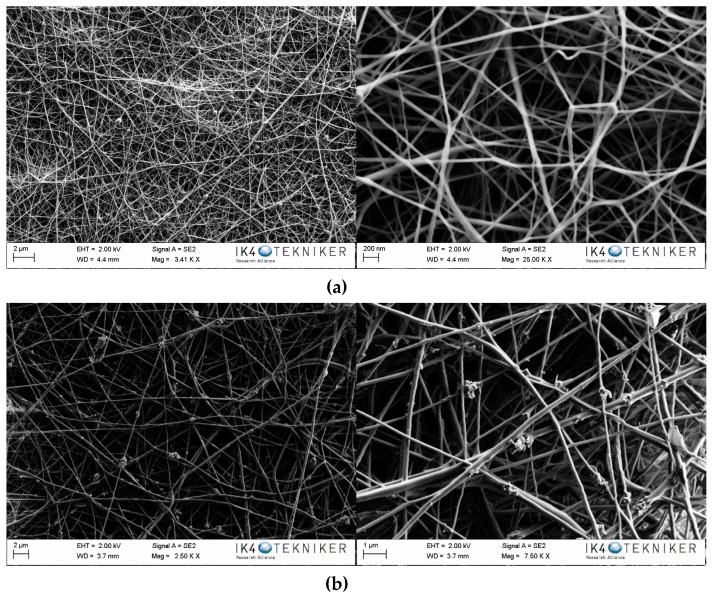
SEM images of (**a**) PA6 and (**b**) PA6/25 wt.% TiO_2_ nanoparticles electrospun nanofibres.

In addition, scanning electron microscopy was used to investigate the morphology and topography of developed membranes. In Figure 3, the SEM images of the electrospun nanofibres membranes made of PA6 and PA6 modified with 25 wt.% of TiO_2_ nanoparticles are shown. The average diameter of the nanofibers and the areal weight of both membranes are summarized in Table 1.

As it is observed in Figure 3a, a continuous and uniform nanofibre membrane without bead formation was obtained using the selected electrospinning working parameters for the PA6 nanofibers. The presence of aggregates of nanoparticles deposited on PA6 nanofibres can be observed in Figure 3b. Considering the high concentration of nanoparticles employed in the developed electrospun nanofibres membranes, it was estimated that most of the nanoparticles were inside the nanofibers, which was corroborated by the significant increase in the diameters of the nanofibres from 60–100 nm for the unmodified system to 110–260 nm for the modified one. Similarly, the areal weight increased from 1.9 g/m^2^ for the unmodified system to 4.3 g/m^2^ for the modified system. The use of this high amount of nanoparticles resulted in some agglomerates in the external part of the nanofibres. However, these aggregated nanoparticles on the nanofibres could provide the membranes with the desired photocatalytic property. 

### 3.2. Evaluation of the Photocalytic Activity of the Membrane

**Figure 4:**
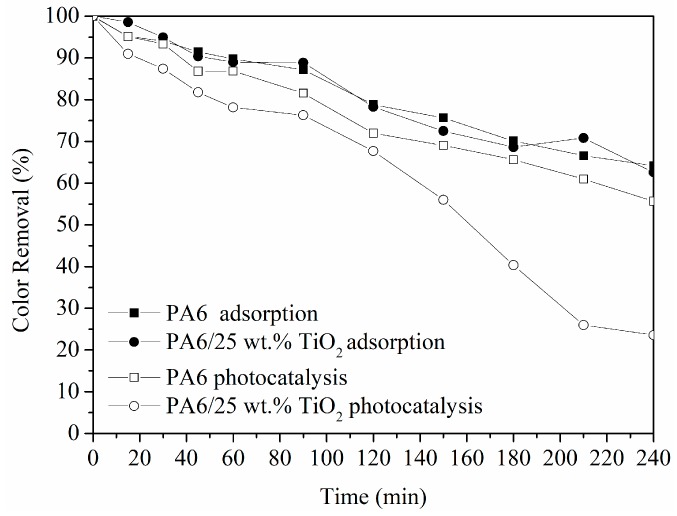
Colour removal of Remazol Black B solution at 597 nm during the contact with PA6 and PA6/25 wt.% TiO_2_ nanoparticles electrospun nanofiber membranes with and without UV light.

**Figure 5:**
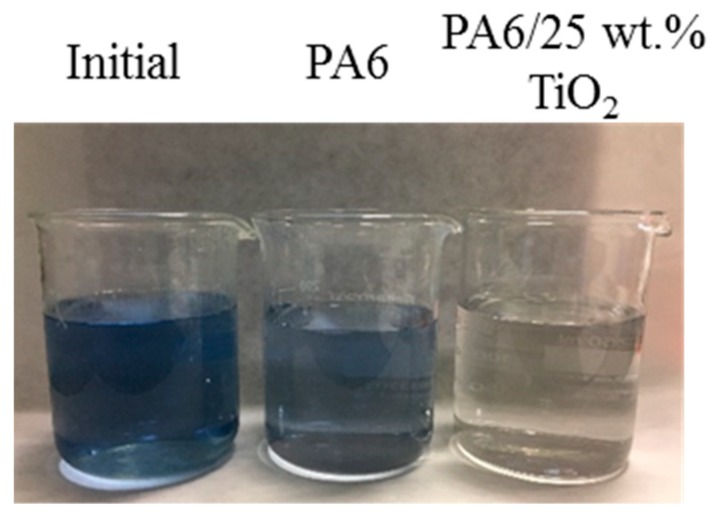
Images of dye solution’s colour (from left to right): initial, after 4 h of contact with PA6 nanofiber membranes under UV light and after 4 h of contact with PA6/25 wt.% TiO_2_ nanoparticles nanofiber membranes under UV light.

Photocatalytic performance of PA6/25 wt.% TiO_2_ nanoparticles electrospun nanofibers membranes has been evaluated by studying the decomposition of a 3 mg/L solution of Remazol Black B as a model pollutant. The photocatalytic process was monitored by measuring the decrease of dye absorbance (at 597 nm) with respect to the irradiation time. Initially, it was found that Remazol Black B remained stable and no decolouration was observed under UV irradiation as no reduction in dye absorbance at 597 nm was observed. The behaviour of a reference sample, PA6 electrospun nanofibers without TiO_2_ nanoparticles, was also monitored under UV radiation as a reference. The results in Figure 4 showed a slight reduction in the absorption band characteristic of this dye at 597 nm, which was related to the adsorption of Remazol Black B on the membrane, since PA6 membrane has no photocatallytic activity. This adsorption capacity was due to the high surface area of the nanofibre nonwoven filter. Similarly, when the modified membrane was not irradiated by UV light, the adsorption capacity for PA6/25 wt.% TiO_2_ electrospun nanofibers membrane was also observed. In this case, after 240 min, the membrane had absorbed a 40 wt.% of the dye. However, the reduction in dye concentration was clearly higher when the system was irradiated with UV light, with half of the dye colour being photocatalytically degraded within 120–150 min. A final 80% of dye was degraded after 240 min, indicating that the phototocatalytic activity of the membrane was due to the presence of TiO_2_. In Figure 5, the variation of the dye concentration after each of the processes under UV light was observed, highlighting the significant contribution of photocatalysis to remove the 2-(4-Aminophenylsulfonyl)ethyl hydrogen sulphate from the solution. 

**Figure 6:**
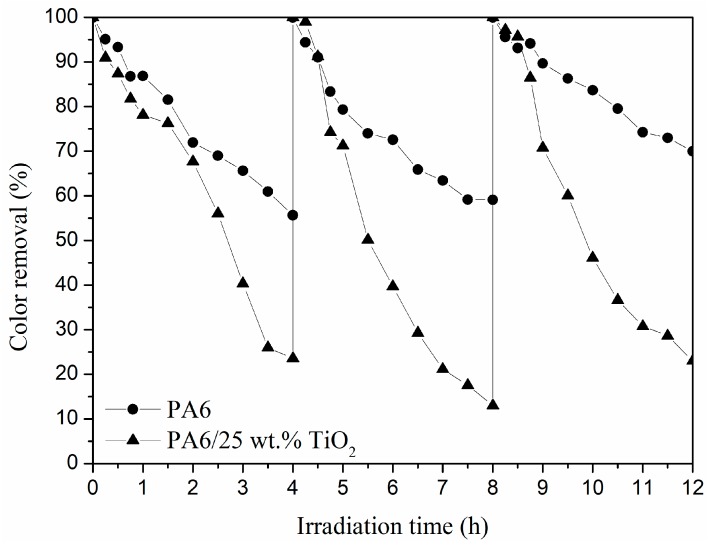
Photocatalytic degradation of Remazol Black B at 597 nm during the consecutive photocatalytic cycles for the PA6 and PA6/25 wt.% TiO_2_ nanoparticles electrospun nanofibre membranes.

Moreover, reusability of PA6 and PA6/25 wt.% TiO_2_ nanoparticles electrospun nanofibre membranes was tested by measuring their photocatalytic activity during three UV lamp irradiation cycles. After each cycle, the materials were placed again in fresh Remazol Black B solution. The results are shown in Figure 6. The results indicated that PA6/25 wt.% TiO_2_ nanoparticles electrospun nanofibre membranes presented good stability and maintained its photocatalytic behaviour after three uses, whereas the PA6 nanofibre membranes displayed a reduced absorption capability over multiple cycles. 

On the other hand, to determine the photocatalytic performance of PA6/25 wt.% TiO_2_ nanoparticles electrospun nanofibres membranes with respect to the removal of *E. coli* and other coliform bacteria present in water, samples of non-modified and modified membranes was put in a glass in contact with 100 mL of artificially inoculated water for 24 h. In some tests, the glasses were irradiated with UV light, and in others, they were kept in the dark. After this period, the water in contact with the membranes was evaluated with CCA Microinstant ® kits in order to determine the presence of *E. coli* and other coliform bacteria (ISO 7704: 1985). The photographs of the filters incubated at 36 ± 2 °C are shown in Table 2. After 96 h of incubation in an oven, most of the filters presented purple-coloured colonies of bacteria, indicating the simultaneous action of the enzymes present in *E. coli* and coliform bacteria. However, the filter that was used to filter the water in contact with PA6/25 wt.% TiO_2_ nanoparticles electrospun nanofibres membranes did not possess any bacteria when the system after being irradiated by UV light. This fact was confirmed via the photocatalytic effect of the membranes. 

## 4. Conclusions

Hybrid nonwoven membranes based on polyamide 6 (PA6) nanofibers modified with a 25 wt.% of titanium dioxide nanoparticles (TiO_2_) were successfully prepared via electrospinning. The targeted decoration of TiO_2_ nanoparticles provided excellent photocatalytic activity of the materials under UV light irradiation. On the one hand, an 80% Remazol Black B degradation was observed after 240 min in the solution in contact with the membrane when this was irradiated by a 365 nm UV light source, indicating the phototocatalytic activity of the membrane. Moreover, reusability of PA6/25 wt.% TiO_2_ nanoparticles electrospun nanofibre membranes was tested during three cycles and maintained its efficiency, whereas the PA6 nanofibre membrane displayed a reduced absorption capability over the cycles. On the other hand, *E. coli* and other coliform bacteria used to artificially inoculate the water were completely eliminated after 24 h of contact with the PA6/25 wt.% TiO_2_ electrospun nanofibre membranes when irradiated with UV light. The presence of these bacteria was not observed even after 4 days of incubation at 36 ± 2 °C. The PA6/25 wt.% TiO_2_ nanoparticles electrospun nanofibre membranes were shown to be reliable for the purification of waters from organic pollutants and for the removal of *E. coli* and other coliform bacteria from water. The new developed membranes were shown to be a suitable and reliable alternative to guarantee fresh water level and quality, mitigating the water scarcity problem worldwide.

## Figures and Tables

**Table 1:** Average diameter and areal weight of the PA6 and PA6/25 wt.% TiO_2_ nanoparticle electrospun nanofibre membranes.

Sample	Average Diameter (nm)	Areal Weight (g/m^2^)
PA6	60–100	1.94
PA6/25 wt.% TiO_2_	110–260	4.30

**Table 2:** Photographs of the filters after 24 h or 4 days of incubation at 36 ± 2 °C.

Incubation Time (h)	PA6 Electrospun Nanofibre Membranes	PA6 Electrospun Nanofibre Membranes	PA6/25 wt.% TiO_2_ Electrospun Nanofibre Membranes	PA6/25 wt.% TiO_2_ Electrospun Nanofibre Membranes
	Without UV	With UV	Without UV	With UV
24	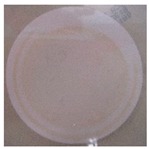	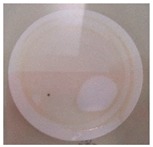	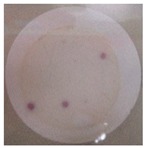	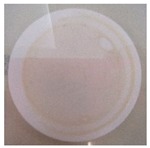
96	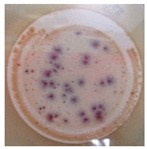	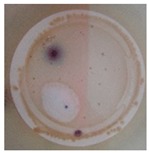	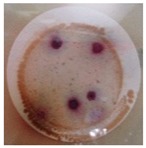	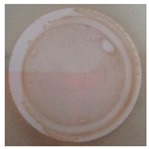

## Supplementary Materials

The following are available online at https://www.mdpi.com/2073-4360/11/5/747/s1.
