# Supplementary material for: TiO2-Doped Electrospun Nanofibrous Membrane for Photocatalytic Water Treatment
Source: Polymers (Basel). 2019 Apr 26;11(5):747. doi: 10.3390/polym11050747 (PMC6572155; doi:10.3390/polym11050747)
Supplement: Supplementary file 1 [file polymers-11-00747-s001.pdf]

Supporting Information

TiO_2 doped electrospun nanofibrous membrane for photocatalytic water treatment

Miren Blanco [1,*], Cristina Monteserín [1], Adrián Angulo [1], Ana Pérez-Márquez [2], Jon Maudes [2], Nieves Murillo [2], Estíbaliz Aranzabe [1], Leire Ruiz-Rubio [3,4] and Jose Luis Vilas [3,4,]

[1] Unidad de Química de superficies y Nanotecnología, Fundación Tekniker, Iñaki Goenaga 5, 20600 Eibar, Spain; miren.blanco@tekniker.es (M.B.); cristina.monteserin@tekniker.es (C.M.); adrian.angulo@tekniker.es (A.A.); estibaliz.aranzabe@tekniker.es (E.A.)

[2] Division Industria y Transporte, TECNALIA, P Mikeletegi 7, E-20009 Donostia-San Sebastian, Spain; ana.perez@tecnalia.com (A.P-M); nieves.murillo@tecnalia.com (N.M), jon.maudes@tecnalia.com (J.M);

[3] Grupo de Química Macromolecular (LABQUIMAC) Dpto. Química-Física, Facultad de Ciencia y Tecnología, Universidad del País Vasco (UPV/EHU), 48940 Leioa, Bizkaia, Spain; joseluis.vilas@ehu.eus (J.L.V)

[4] BCMaterials, Basque Center for Materials, Applications and Nanostructures, UPV/EHU Science Park, 48940 Leioa, Spain

* Correspondence: miren.blanco@tekniker.es; Tel.: +34-943-20-67-44

For the PA6/TiO_2 electrospun nanofibres membranes preparation, a 12 wt% of PA 6 Ultramid pellets were dissolved in the mixture 2:1 acetic acid:formic acid by stirring during 2 hours at 80 ºC. TiO_2 nanoparticles were added to the PA6 solution obtaining a system with 25% by weight of nanoparticles with respect to PA6. The stabilization of the nanoparticles requires the addition of a surfactant.

Two types of surfactants, polyethylene glycol (PEG) and sodium dodecyl sulphate (SDS) in a 1:1 weight ratio with respect to nanoparticles weight were explored. Both surfactants dissolve completely in the solvent mixture of the dispersion. The systems prepared with both surfactants were mechanically stirred for 30 min, and then sonicated in a US Sonics VCX series with a power of 750 W and amplitude of 30%. The stability of the dispersions was determined by Dynamic Light Scattering (DLS) by using a Z sizer Nano from Malvern. Samples were taken during the sonication steps, when the samples have been submitted to 30 min or 60 min of sonication and also, 1 h after stopping the sonication.

For the DLS analysis, a dilution of the samples has been carried out since they are too concentrated. Figure S1 shows the DLS curves obtained for the TiO_2 dispersions using both surfactants at the different sonication times. For each analysis, 3 measurements were made, and the particle size value was taken as an average of the three (Table S1).

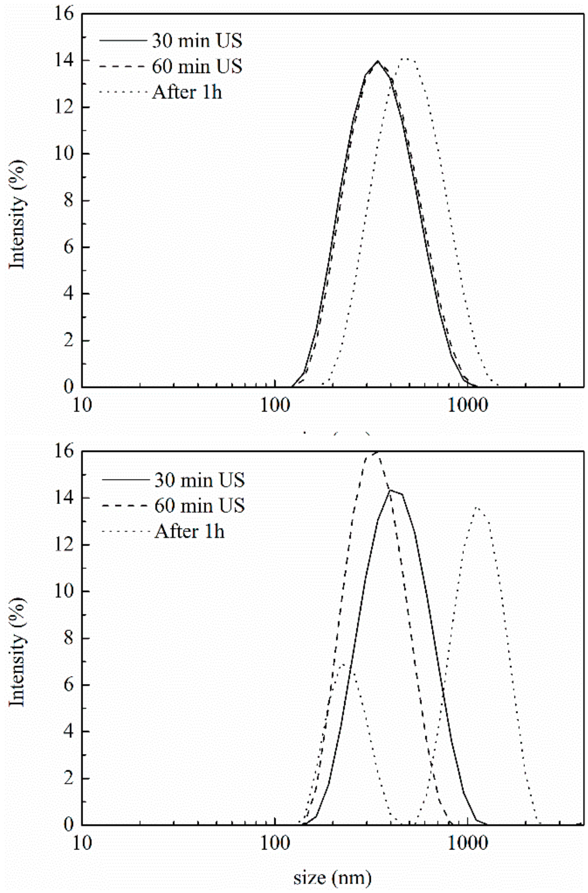

Figure S1. Intensity size distribution of TiO_2 nanoparticles versus particle diameter in nanometers for the dispersions prepared using both surfactants: a) SDS and b) PEG, at the different sonication times.

Table S1. Values of average particle size obtained by DLS measurements for the dispersions prepared using both surfactants: a) SDS and b) PEG, at the different sonication times.

**Ultrasound time**	**SDS**	**PEG**
	**Average size (nm)**	**Average size (nm)**
30 min US	322.4	379.4
60 min US	332.7	339.3
60 min ultrasounds+1 h repose	358.2	734.5

Using SDS it is observed that the average values of the agglomerates are similar after applying 30 or 60 min of ultrasound. 1 hour after the ultrasound process, a small aggregation is observed that is not significant since large agglomerates do not appear above the micron and most of the nanoparticles are in the same range of size distribution. Therefore, it can be considered that the dispersion is stable during the electrospinning process, which lasts less than 1 h.

In the case of the PEG, it is observed that 1 h of ultrasound process is necessary to reduce the agglomerate size as much as possible. In addition, a greater aggregation of the nanoparticles is observed during the repose time, 1 hour after the ultrasound process. A large mode appears at sizes above the micron, so it can be considered that the dispersion would not be stable during the electrospinning process and the use of this surfactant has been ruled out.

As a consequence, it has been selected to apply 30 min of ultrasounds using the SDS as a surfactant for the preparation of the TiO_2 nanoparticle dispersion in the precursors of the membranes.
